# Editorial: The effects of chemotherapy towards the tumor microenvironment

**DOI:** 10.3389/fonc.2022.1069561

**Published:** 2022-11-01

**Authors:** Nadiah Abu, Alice Turdo, Jose A. Garcia-Sanz

**Affiliations:** ^1^ Universiti Kebangsaan Malaysia (UKM) Medical Molecular Biology Institute (UMBI), National University of Malaysia, Bangi, Malaysia; ^2^ Department of Health Promotion, Mother and Child Care, Internal Medicine and Medical Specialties (PROMISE), University of Palermo, Palermo, Italy; ^3^ Centro de Investigaciones Biologicas Margarita Salas (CIB-CSIC), Department of Molecular Medicine, Consejo Superior de Investigaciones Cientificas, Madrid, Spain

**Keywords:** chemotherapy, tumor microenvironment, immunogenic, cancer, therapy

Chemotherapy is the first-line therapy for cancers lacking tailored targeted therapies. Nevertheless, the efficacy of cytotoxic compounds is severely compromised by intra- and inter-tumor heterogeneity and most importantly by the presence of pro-tumorigenic cells and factors belonging to the tumor microenvironment. A comprehensive review reported on this Research Topic embraces multiple insights regarding the resistance of cancer cells to chemotherapeutic drugs (Gaggianesi et al.). Compelling evidence demonstrates that the resistance to chemotherapeutic agents is sustained by the presence of a subpopulation of stem-like cells, named cancer stem cells, endowed with tumor-initiating and metastatic capabilities. The studies listed by Gaggianesi et al. attribute a fundamental role to tumor microenvironment in dictating cancer stem cells refractoriness. The most described mechanisms are related to the upregulation in the expression of drug efflux transporters, anti-apoptotic proteins and the activation of survival pathways (Gaggianesi et al.). In ovarian cancer, chemotherapy also affected cancer stem cell gene expression. (Escalona et al.). The study by Escalona et al. showed that platinum-based therapy altered the expression of several MMP and TIMP genes in association with some cancer stem cell-related genes.

Although chemotherapy is ineffective against the subpopulation of cancer stem cells, pivotal studies reviewed by Garofalo et al. describe how chemotherapy positively affects the tumor microenvironment by restoring the natural killer (NK) cell’s functions. One of the most important features of cytotoxic compounds is to increase the immunogenicity of cancer cells improving their recognition by NK cells within the tumor microenvironment (TME). From a different perspective, the latter event is also facilitated by an augmented capability of NK cells to penetrate the tumor microenvironment following chemotherapy administration. A plethora of mechanisms including the secretion of chemoattractant cytokines and changes in collagen fibers composition have been postulated. Garofalo et al. thoroughly discussed the advantage of using nanocarriers loaded with chemotherapy compounds to yield a targeted drug delivery. Even though the findings provided by Garofalo et al. are focused on melanoma cells, the presence of natural killer in TME correlates with the outcome in a variety of cancers, thus making these findings apply to a large number of cancer cases. Altogether, these achievements could also have a huge throwback in clinical practice by widening the use of chemotherapy in patients refractory to immunotherapy. Apart from that, a review by Di Ianni et al., also focused on the immunomodulatory effects of temozolomide (TMZ) in glioblastoma, especially in relation to TME. The authors reviewed that the paradoxical role of TMZ as a chemotherapeutic agent may produce an immunosuppressive environment instead. Thus, the role of TMZ as an immunological adjuvant remains questionable.

Damage-associated molecular patterns (DAMPs) are immune-stimulating molecules that are released by cells upon cell death or cell stress and can be caused by chemotherapy. One of the DAMP molecules is the HMGB-1 protein, as highlighted by Chavez-Dominguez et al. The dual roles of HMGB-1 in relation to platinum-based treatments have been controversial. Nevertheless, the authors have demonstrated that there is indeed a correlation between the levels of HMGB-1 in the plasma of the patients with increased overall survival of lung adenocarcinoma patients undergoing cisplatin treatment.

In an original article, Liu et al. analyze the effects of the tyrosine kinase inhibitor cabozantinib *in vivo* using a mouse model of renal cell carcinoma. The authors show that this inhibitor increased the secretion by the tumor microenvironment of chemokines involved in the migration of neutrophils and CD8+ T cells, leading to an increase in tumor infiltration by these cells and a decrease in tumor growth. Conversely, the depletion of neutrophils and CD8+ T cells in the animals significantly decreased the anti-tumoral effect of the inhibitor. Furthermore, cabozantinib treatment induced long-term anti-tumor T-cell responses, suggesting that cabozantinib might represent a good candidate for combination therapies, not only with chemotherapy but also with T-cell therapies or other immunotherapies.

Naseer et al. review the advanced therapeutic innovations to improve survival and reduce morbidity and mortality in metastatic castration-resistant prostate adenocarcinoma. Interestingly, these innovations take into account most of the biological features of prostate cancer. The microenvironment in this type of tumor is highly relevant since the elevated levels of prostate-specific antigen (PSA) lead to increased androgen receptor (AR) levels and their associated signaling pathways. They discuss the approaches to use steroidogenic enzymes to inhibit testosterone and dihydrotestosterone levels, and inhibitors of the androgen synthesis pathway (abiraterone, enzalutamide, andapalutamide). but also, the targeting of AR-independent signaling pathways, focusing on DNA damage repair overcoming mechanisms including inhibitors of Poly(ADP–ribose)polymerase (PARP), as well as a whole plethora of gene-based therapies and discuss recent data on new chemotherapeutic options. In addition, they also review data on the use of the anti-immune checkpoint antibody anti-PD-1 (pembrolizumab), to burst the patient’s immune response to tumoral antigens. Taken together, these data point towards a changing therapeutic profile for advanced prostate cancer that might lead to substantial changes in survival.

We believe that this Research Topic has been able to collect research that highlights and provides insights into the effects of chemotherapy on the tumor microenvironment, as summarised in **Figure 1**, and hopefully points toward the future line for research in the field.

**Figure 1:**
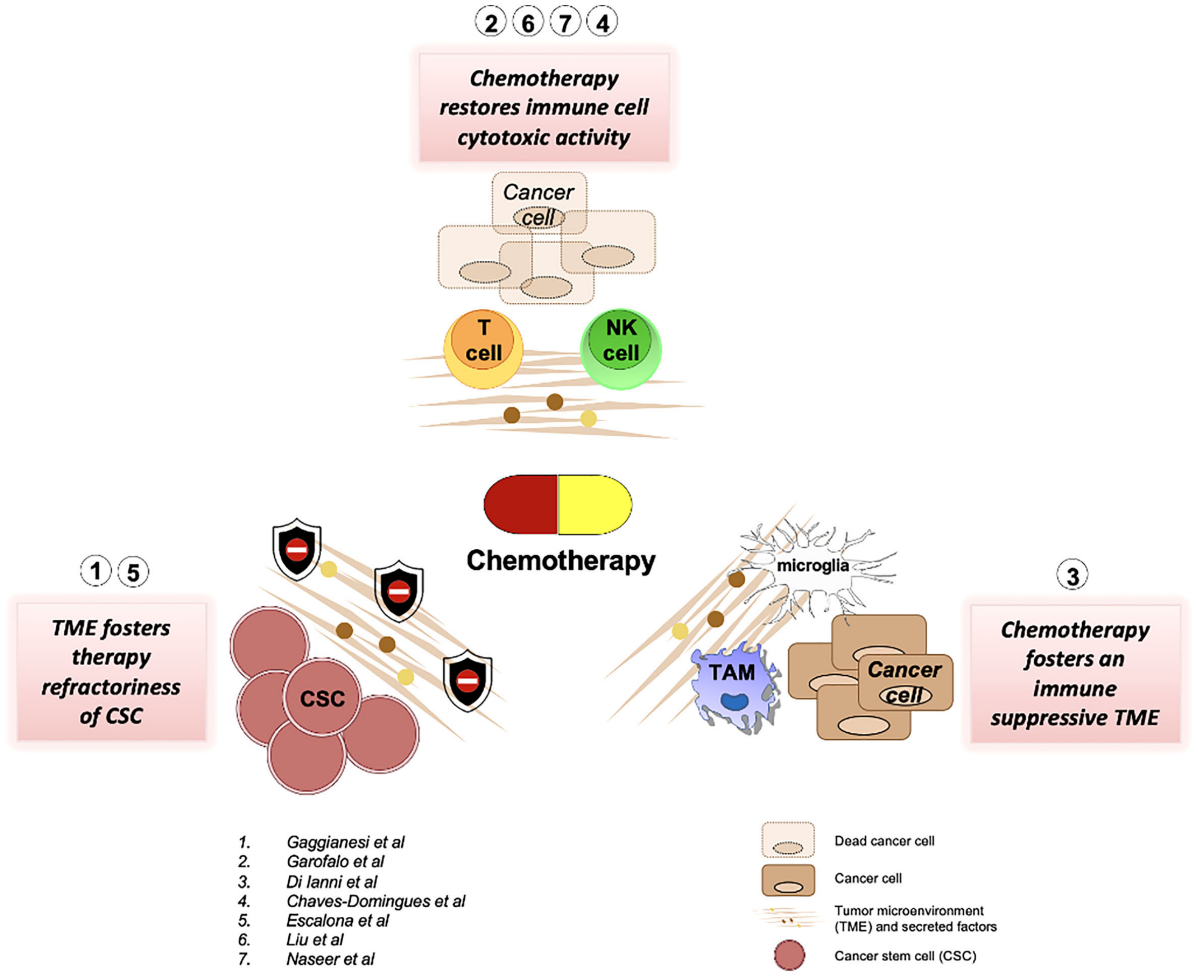
A collective summary of the articles published in this research topic.

## Author contributions

NA, AT and JG-S designed and wrote this editorial article. All authors contributed to the article and approved the submitted version.

## Funding

The work in the author’s laboratories was partially supported by grants from the Spanish Ministry of Science and Innovation (PID2019-105404RB-I00 financed by MCIN/AEI/10.13039/501100011033) to JAGS.

## Acknowledgments

If this topic is successful, it would reflect the author’s effort, the excellent work of reviewers and external editors, and the Frontiers Editorial Office invaluable help. We would like to acknowledge all of them for their enthusiasm. We hope that this Research Topic will foster future international collaborations among the authors and with other researchers in the field.

## Conflict of interest

The authors declare that the research was conducted in the absence of any commercial or financial relationships that could be construed as a potential conflict of interest.

## Publisher’s note

All claims expressed in this article are solely those of the authors and do not necessarily represent those of their affiliated organizations, or those of the publisher, the editors and the reviewers. Any product that may be evaluated in this article, or claim that may be made by its manufacturer, is not guaranteed or endorsed by the publisher.

